# Eating Out and Consumers’ Health: Evidence on Obesity and Balanced Nutrition Intakes

**DOI:** 10.3390/ijerph17020586

**Published:** 2020-01-16

**Authors:** Dahye Kim, Byeong-il Ahn

**Affiliations:** 1 Department of Agricultural, Food, and Resource Economics, Michigan State University, East Lansing, MI 48823, USA; kimdahye@msu.edu; 2 Department of Food and Resource Economics, Korea University, Seoul 02841, Korea

**Keywords:** food away from home, balanced dietary intakes, obesity, doubly robust estimation, inverse probability weighted regression adjustment

## Abstract

Changes in demographic and socioeconomic characteristics have contributed to an increase in away-from-home food consumption. Although consumers are increasingly demanding higher quality food, unbalanced nutrition intakes and health issues such as obesity remain prominent predicaments. This paper investigates the relationship between the frequency of having Food Away From Home (FAFH), balanced dietary intakes, and obesity (controlling for covariates) among Korean adults aged 19 to 64. Whether there exists a linear relationship between the number of having FAFH and health outcome is investigated and the optimal number of having FAFH that leads to the best health outcome is identified in the study. The results suggest that Food Away From Home generally increases deviations of dietary intakes from the reference intakes and high-frequency FAFH consumers have an elevated chance of being obese (36.22%). However, having FAFH 1–7 times per week is associated with decreased body mass index (BMI) and a lower chance of being obese in comparison to the outcomes of having food at home. The optimal level of consuming FAFH is identified to be 5–7 times per week in terms of BMI and obesity. However, consuming no FAFH is suggested to be the best in terms of balanced nutrition intake.

## 1. Introduction

Changes in demographic and socioeconomic factors have contributed to changes in food consumption behavior. For example, women’s participation in the workforce and the increased opportunity cost of time have caused people to demand more convenient food products [1,2]. Falling household sizes and the increase in the number of single households have led to a reduction in the economies of scale of meal preparation at home [3]. As a result, the consumption of processed food and away-from-home foods has significantly increased and caused concerns about people’s health [3].

Consumers’ nutrient intakes and overall health can be affected by where and what they eat [4,5,6]. Away-from-home foods are generally linked to more adverse health outcomes compared to at-home foods [7,8,9]. As eating habits are closely related to health, it is crucial to intake all of the required nutrients right amount in order to have a balanced diet and maintain good health status. While the relationship of health outcomes to away-from-home food consumption has been explored, a more comprehensive investigation on this type of consumption and its relation to health effects specifically driven by inadequate nutrient intake is required.

Food Away From Home (FAFH) is defined as purchased meals and snacks prepared by commercial foodservice establishments and eating facilities in non-commercial institutions [10]. Commercial foodservice includes limited-service restaurants such as cafeterias, quick service restaurants, full-service restaurants, bars, hotels, retail stores, and recreational places such as movie theaters. Many researchers have explored the effect of FAFH on diet quality and health outcomes. For example, eating one meal away from home each week is known to increase daily caloric intake, leading to a negative effect on diet quality for the average adult [11]. Larson et al. [12] analyzed the effect of FAFH by type of restaurants on dietary intake and weight status among young adults. It was found that frequent fast-food consumers are associated with a higher risk of being overweight/obese and having a poorer diet quality, whereas the frequent full-service restaurant consumers are not associated with being overweight. However, on the other hand, the result of Binkley [13] indicated that there is no significant difference between meals from table service and fast food in terms of caloric intake even though both foodservice meals have caloric intakes higher than at-home meals. Prescott and Logan [14] pointed out that wealthy people’s health outcomes, which are associated with visiting fast-food outlets, differs from the outcomes of poor consumers. Zagorsky and Smith [15] found that adults in the highest quintile of income are less likely to report fast-food consumption than those in the lowest quintile, and adults in the second and third quintiles are more likely to report fast-food consumption than the poorest.

According to the World Health Organization, obesity is defined as abnormal or excessive fat accumulation that presents a risk to health. The main measurement of obesity is body mass index (BMI) which is calculated as a division of a person’s weight by the square of height. When BMI is equal to or greater than 30, the person is considered obese. Bezerra et al. [16] studied the association between eating away from home and being obese and found that FAFH is not associated with obesity status among men and is negatively related to being overweight and obese among women for the average Brazilian living in an urban area. However, away-from-home food consumers had higher intakes of energy-dense foods. Naska et al. [17] found that individuals who consume equal to or greater than 25% of their daily energy intakes away-from-home are similar with at-home eaters in terms of food intakes. However, those who consume less than 25% of their daily energy intakes out-of-home are different from at-home eaters: they consume more food such as sweets, savory baked goods, and non-alcoholic beverages and less of meat, seafood, and vegetables.

Few previous studies on this topic have used data from Asian countries. Du et al. [18] studied gender differences in the effect of FAFH on body weight outcomes among Chinese adults. The study indicated that FAFH increases BMI and waist circumference, and FAFH is more likely to affect males rather than females. Kim et al. [19] found that heavy FAFH consumption (taking more than 1400 kcal from FAFH on a single day) is related to a higher BMI and waist circumference. Park et al. [20] researched the impacts of FAFH on caloric intakes: their results showed individuals who consume FAFH at least once a day have higher caloric intake in comparison to individuals who have meals at home. Kim [21] demonstrated that consuming away-from-home foods that are high in calories increases BMI although the magnitude is small. 

In most of these studies, simply the effect of binary treatment of whether or not consuming FAFH has been analyzed. However, the health outcomes of consumers who depend heavily on FAFH would not be similar to consumers who consume FAFH less frequently, but still depend on it as a food source. In this regard, it is worth while to investigate whether there exists a linear relationship between the frequency of consuming FAFH and the degree of health outcome such as degree of obesity. If there exists a linear relationship, it implies that the effects of having one more FAFH meal on health outcomes are the same regardless of whether the consumer of interest already heavily depends on FAFH or not. While prior studies such as Bhutani et al. [22], Todd et al. [11], Paeratakul [23], Marín-Guerrero et al. [24], Smith et al. [25], Ayala et al. [26], Orfanos et al. [27] and Cornelisse-Vermaat and van den Brink [28] have explored the effects of FAFH in the context of linearity between FAFH and health outcomes, few studies have addressed the issue of none-linearity between FAFH and health outcomes. For example, Boutelle et al. [29], Fulkerson et al. [30], Seguin et al. [31] and McClain [32] divided consumers into several groups according the frequency of consuming on FAFH and effects of FAFH on BMI are estimated separately for each consumer group. In these studies, it was found that the marginal effect of eating out once more for the consumer who depends heavily on FAFH differs from the marginal effect for the consumer who consumes FAFH less frequently, which shows the existence of non-linearity. This study tries to address a bit deeper research question regarding the non-linearity. If marginal effects of FAFH on health outcome appear to not be linear, it may comprise positive to negative (or negative to positive) marginal effects, which was not manifested in the prior studies ([29,30,31,32]). In this sense, the subsequent research question that deserves to be investigated is the optimal level of frequency of having FAFH that results in the best health outcome such as minimizing obesity. 

Focusing these two research questions, the present study explores the effects of the frequency of consuming FAFH on balanced nutrition intake and the corresponding health outcome of obesity. In other words, multi-level hypothetical treatments of the frequency of FAFH are investigated in this study. Prior studies that have shown non-linear relationship between frequency of having FAFH and health outcomes estimate the effects by applying a regression model for consumer groups divided by FAFH frequency (Fulkerson et al. [30], Seguin et al. [31] and McClain [32]). Therefore, self-selection bias remains an issue. This study aims to fill this gap by utilizing a treatment effect model. In the empirical analysis of this study, the inverse probability weighted regression adjustment, a doubly robust method for eliciting multiple treatment effects, is applied to the case of South Korea which shows unique characteristics of food consumption due to a rapid economic development in a very short period of time. 

The remainder of this paper is organized as follows: Section 2 and Section 3 describe the dataset and methodology used in the present study, respectively. Section 4 presents the estimation results. Section 5 concludes with some policy implications.

## 2. Data

In terms of quantity as well as quality, food consumption patterns in South Korea have changed greatly during the last several decades with rapid economic growth. For example, the daily caloric intake of a South Korean consumer has increased from 2876 Kcal in 1990 to 3128 Kcal in 2016. Additionally, the proportion of fat intake among the total intake of three essential nutrients (carbohydrates, protein, and fat) was 14.1 percent in 1990 and increased to 21.6 percent in 2014. As a result, imbalanced nutrition intakes and obesity have become social issues, as clearly indicated by the percentage of being obese, which has been increased from 26.0 percent in 1998 to 34.8 percent in 2016. According to the Harvard School of Public Health, consuming a higher or lower proportion of any of the three essential nutrients than the required intakes can lead to malnutrition and poor immune system. As for the economic effect of these adverse health outcomes, the Korean National Health Insurance Service estimated the cost incurred by imbalanced nutrition intake and corresponding obesity as $9.88 billion. 

**Figure 1:**
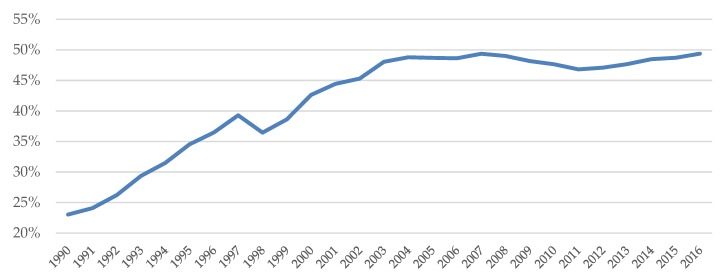
Food Away from Home as a share of total food expenditures. Source: Statistics Korea Household Income and Expenditure Survey.

There are many possible explanations for the obesity of adults. However, in Korea, Food Away From Home has drawn attention [14,20,21,22]. Figure 1 presents the trend of FAFH as a share of total food expenditures in Korea. Over the past three decades, the share of FAFH has increased significantly, from about 23% in 1990 to 50% in 2016. This is a very similar pattern of the change in the percentage of the South Korean population that is obese. 

We can see comparable changes in food consumption patterns such as an increase in calorie intake, in many Asian countries, especially in China and countries in the southeastern part of the continent. These countries currently show a very high rate of income growth similar to South Korea during the last decades. Therefore, Korea’s relationship between food consumption and health outcomes would be a good reference for these countries. Therefore, this study has selected Korea as the target of its analysis. 

The raw data used in this research is the sixth Korean National Health and Nutrition Examination Survey (KNHANES VI) from 2013 to 2015. The survey is conducted by Korea Centers for Disease Control and Prevention annually with a rolling survey sampling method and the survey data represents the population in Korea. In the sixth KNHANES (2013–2015), 29,321 participants from all age groups were surveyed. Of the 29,321, 22,948 individuals participated in at least one health interview, health examination, and nutrition survey. The total number of the sample utilized in this study is 7456 after excluding the data from those who did not respond to the questions fundamental to this analysis (this is free public data provided by the government, therefore there is no need for ethical approval). 

In the KNHANES, individuals were asked “On average, how often did you consume away-from-home foods (including delivery meals, take-out foods, provided meals from school or religious service, etc.) over the past year?”. Individuals who consume provided meals from school, work, religious services or other organizations are excluded in the study samples since they generally have been guided by a nutritionist. Thus, the results could be confounded if they are included in the analyses. 

In this research, the frequency of consuming FAFH is considered to analyze the effect of each different level of having FAFH consumption, which is set as a different level of hypothetical treatment in the empirical analyses, on the health outcomes. Table 1 describes the multivalued variable of FAFH. The value of variable takes on 0, 1, 2 and 3 depending on the frequency of having FAFH.

**Figure 2:**
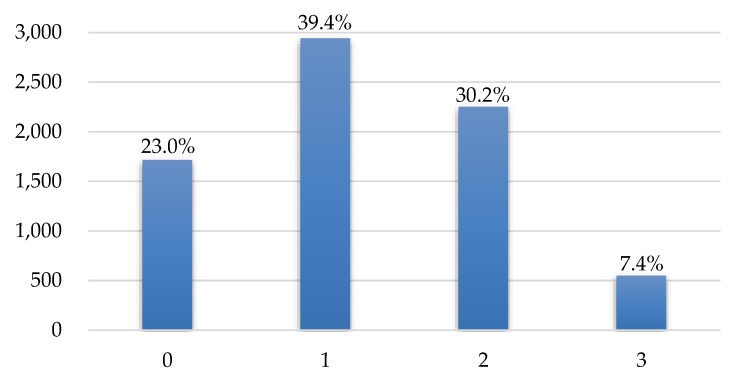
The number of participants in each FAFH category.

Figure 2 shows the number of participants in each category of FAFH frequency level. 2941 participants in category 1 consume FAFH 1–4 times per week, 2251 participants in category 2 have FAFH 5–7 times per week, and relatively less proportion of participants (23%) have FAFH at least twice a day. Participants who mostly have food at home are in category 0 which is considered the reference group. 

Dietary intakes data for each respondent can be obtained from 24-h recall intakes (food intakes consumed during the past 24 h are surveyed) or Food Frequency Questionnaire (the Food Frequency Questionnaire surveyed the frequency of food intake and intakes per serving over 112 food items, over the past year) in the nutrition survey. Many previous studies used 24-h dietary recall data by assuming that the dietary intakes represent the long-term dietary habits at an individual level. However, this assumption could overstate or understate the estimated results if the data does not represent a regular diet for each individual. In the present research, dietary intakes data from the Food Frequency Questionnaire, which was surveyed for 11,264 adults aged from 19 to 64, is used because it has a comparative advantage over the 24-h recalls. 

Table 2 summarizes the descriptive statistics of the outcome variables used in this empirical analysis. Chosen outcome variables are energy, protein, fat, carbohydrate, three essential nutrients, calcium, sodium and potassium intakes, body mass index and obesity dummy variable. 

Every individual requires a different amount of nutrients and thus has different reference for each nutrient. In order to analyze the effects of FAFH on the balanced dietary intakes, absolute values of the percentage deviation of each daily dietary intake from the corresponding reference intake are calculated (2015 Dietary Reference Intakes for Korean from Ministry of Health and Welfare provides the reference intakes of nutrients. (Table S1). For the three essential nutrients intakes, the average of the absolute values of the percentage deviation of protein, fat, and carbohydrate is calculated. When the daily dietary intakes are close to the reference intakes, the absolute values of the percentage deviation will be close to zero, which means that the nutrition intakes are relatively balanced.

It can be observed that sodium intakes are far from adequate intake level as the mean of “percentage deviation of sodium” is 1.26 whereas energy intakes are relatively more balanced (the mean of “percentage deviation of energy” is 0.27). The average body mass index (BMI) is 23.68 which is in the range of normal weight (18.5 to 25) and 31.14% of the study population has BMI greater than or equal to 25 kg/m^2^ which is defined as overweight.

To elicit the effects of having FAFH, covariates need to be controlled for. Table 3 describes the summary statistics of covariates, which are selected based on prior research that investigated the determinants of food consumptions. Socio-economic characteristics and health related variables are controlled in the analysis as these factors influence the demand for FAFH [13,22,23,24,25,26]. 

Of the sample, 38% are male; the average age is 44; 47% live in urban areas; 40% have educational level higher than or equal to university graduation; the average number of family member is 3.24; 6% are single households; the average monthly household income is about 4,157,800 KRW; 80% are married; 41.6% have at least one child; and 18.8% and 23.8% have full-time and temporary jobs, respectively. Those who are in subjectively good health status account for a third of the study sample. 46% have subjective body perception (Seo et al. [33] demonstrated that subjective body perception influences food intakes and weight control) as fat and 26% experience high levels of stress and 21% drink alcohol twice or more per week. Respondents who have high blood pressure and pre-hypertension are about 20% and 24%, respectively. 

The average number of days of weight training exercise and flexibility exercise per week are 0.78 and 1.91, respectively. Respondents have an average of 2.53 meals per day and 24% of the respondents practice dietetic therapy and 31% of them use nutrition labels when they purchase foods (Kim et al. [34] found the significant effect of food label use on nutrition intakes). These strengths, stretch, and dietetic therapy variables are included in the empirical estimation.

## 3. Methodology

There are plenty of program evaluations reported in the literature estimating average treatment effects in the setting of a binary treatment. For example, Rubin [35] has developed a counterfactual framework that is adopted in the fields of statistics and econometrics. Heckman et al., [36] evaluated a job training program and Schultz [37] studied the effect of providing school subsidies for the poor on enrollment. More studies are found in the social and health sciences (see, for example, Imai and Van Dyk [38], Gibson-Davis and Foster [39] and Foster [40]).

The present research evaluates the effect of FAFH by applying a multivalued treatment approach which is introduced by Imbens [41] and Lechner [42], in order to avoid selection bias. In the present study, the level of frequency of having FAFH is taken into account as it is multivalued in nature. Multivalued treatment effect analysis provides additional information compared to a binary treatment model and allows us to capture the different effects of each treatment levels [43,44,45,46,47]. 

The basic setup of estimating multivalued treatment effects is as follows [47]. Let the variable ω be a multivalued treatment indicator. Then the variable $\omega_{i}$ can take values from 0 to *J*. That is, $\omega_{i}=\left\{0,1,\ldots ,J\right\}$ for *J + 1* treatment level. Each individual, *i*, is in one of the states according to a different level of treatment. If we denote a potential outcome as $y_{ij}$ for the individual *i* in the treatment level *j*, the average treatment effects of *J*-th treatment in comparison to the base treatment (i.e., at the treatment level 0 or no treatment) can be written as $\tau_{J,\mathrm{ate}}\equiv E\left(y_{J}-y_{0}\right)$. However, there arises a missing data problem in estimating this equation since there is only one observed outcome for each individual. In other words, the observed outcome $y_{i}$ for the individual *i* at the *J*-th treatment level can be expressed as Equation (1):

(1) $$y_{i}=\sum^{}d_{i}\left(j\right)\left[\omega_{i}=j\right]y_{ij}=0\left[\omega_{i}=0\right]y_{i0}+0\left[\omega_{i}=1\right]y_{i1}+\cdots +1\left[\omega_{i}=J\right]y_{iJ}$$

where $d_{i}\left(j\right)\left[\omega_{i}=j\right]$ is the indicator function that takes value 1 when the individual *i *receives treatment *j* and 0 otherwise. This means only one potential outcome $y_{iJ}$ is observed for each individual, although other counterfactual outcomes ($y_{i0}$, …, $y_{iJ-1}$) are required to be calculated as average treatment effects on a different treatment level, which we call as missing data problems. This issue can be reflected in the empirical analysis by employing two assumptions.

The first assumption is conditional mean independence (ignorability of treatment) introduced by Rosenbaum and Rubin [48]. The conditional mean independence (CMI) assumption implies that after conditioning on covariates, the means of the potential outcomes are not affected by the treatment (i.e., $E\left(y_{i}|x,\omega \right)=E\left(y_{i}|x\right)$). This assumption holds if there is enough information available to determine treatment status and covariate factors x are not affected by ω. Another assumption required to identify the unconditional average treatment effect is overlap assumption. This assumption means there is a chance to find each individual in the control or treatment groups. The conditional probability of receiving a particular level of treatment, given x, is called as the generalized propensity score (Imbens [41]). This assumption allows us to estimate an average treatment effect over the population with x by adjusting for the propensity score.

In this paper, the hypothetical multivalued treatment effects are estimated by the inverse probability weighted regression adjustment (IPWRA) method. IPWRA method solves the missing-data problem by assigning inverse probability weights to estimate outcome-regression parameters. The IPWRA estimators have doubly robust property since the treatment model (weighting approach) and the outcome regression model (regression adjustment approach) are combined in the estimation to provide consistent parameter estimates even if only one of the models is correctly specified [40,48,49,50] This method is advantageous since the chances of getting consistent parameter estimates are higher. The efficiency costs are almost zero to protect the model misspecification by using the doubly robust method in multivalued treatment effect estimation [50].

We follow two steps in the empirical implementation (The estimation process is following STATA Treatment-Effects Reference Manual Release 13 and Wooldridge [47]). First, we estimate the parameters ($\hat{\gamma }$**)** of treatment model (multinomial logit) to obtain propensity score weights, $p_{j}\left(x_{i},\hat{\gamma }\right)$, where j=0,1,…,J, then regress weighted regression models of the outcome for each treatment level as follows:

(2) $$\underset{\alpha_{j},\beta_{j}}{\min}\sum_{i=1}^{N}\omega_{i}{\left(y_{i}-\alpha_{j}-x_{i}\beta_{j}\right)}^{2}/p_{j}\left(x_{i},\hat{\gamma }\right),$$

where j=0, 1,…,J. 

The second step is the estimation of the average treatment effects (ATEs) by contrasting the averages of treatment-level predicted outcomes as Equation (3):

(3) $${\hat{\tau }}_{J,ate}=N^{-1}\overset{N}{\underset{i=1}{\sum }}\left[\left({\hat{\alpha }}_{J}+x_{i}{\hat{\beta }}_{J}\right)-\left({\hat{\alpha }}_{0}+x_{i}{\hat{\beta }}_{0}\right)\right]$$

## 4. Results

### 4.1. Estimation Results for Treatment Model

This section explores how the conditional probabilities of having different levels of the frequency of FAFH given the observed covariates are estimated by the multinomial logit. Table 4 presents the estimation results for the treatment model by multinomial logit. From the estimation results, the characteristics that have significant positive effects, controlling for other covariates, on the probability of having FAFH are the following: being male, being young, living in urban area, having a high educational level, having small number of family members, living in a single household, earning a higher average monthly household income, being unmarried, having child under 19, being employed, having parents with high educational levels, being subjectively healthy, having no activity limitation, being subjectively fat, frequently drinking, having high stress, having normal blood pressure, not using labels to determine food consumption choices, and having a low number of meals per day.

### 4.2. Covariate Balance Summary

In order to obtain the effects of treatment, the distribution across treatment and comparison groups should be similar. Table 5 presents the baseline means for control and hypothetically treated groups. It can be observed that the means of each covariate significantly vary across groups, especially for sex, age, education level, average monthly household income, marital status, employment type, and drinking frequency. This implies that the covariates need to be balanced by weighting in observational data such that the outcome is independent of the hypothetical treatment after conditioning on covariates.

A covariate is said to be balanced when the standardized difference is zero and the variance ratio is one (Standardized difference is the difference in means standardized by a measure of dispersion, not the t-statistic for testing the difference in means [49]). Tables S2–S5 in the Supplementary Material reports covariates balance summary over the hypothetical treatment groups after inverse-probability weighted.

**Figure 3:**
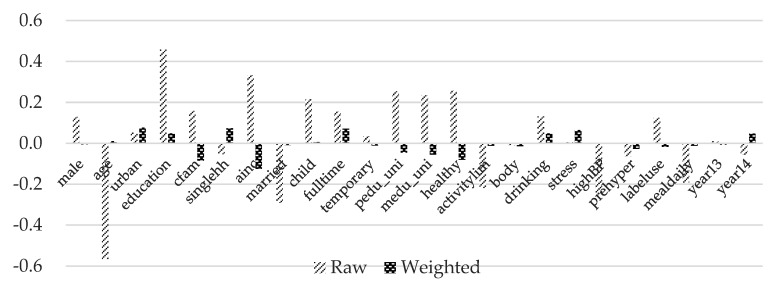
Standardized mean differences of treatment group 1. Note: Detailed explanations of the variables are included in Table 3.

**Figure 4:**
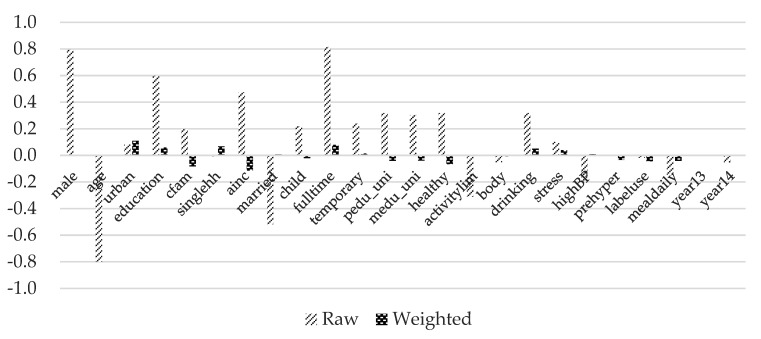
Standardized mean differences of treatment group 2. Note: Detailed explanations of the variables are included in Table 3.

**Figure 5:**
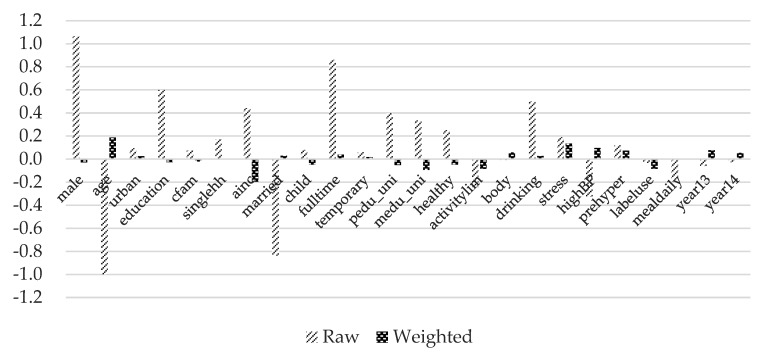
Standardized mean differences of treatment group 3. Note: Detailed explanations of the variables are included in Table 3.

The variance ratios are all close to one except for average monthly household income. Figure 3, Figure 4 and Figure 5 show standardized mean differences between the control group and each treatment group graphically. Imbens and Rubin [50] provides a guideline for the standardized difference between hypothetically treated and controlled situations. They suggested that standardized differences above 0.25 are troublesome. It can be observed that the standardized mean differences are significantly reduced and all below the threshold value of 0.25 after inverse-probability weights are assigned. These results indicate that covariates are well balanced across the hypothetical control and treatment groups.

### 4.3. The Average Treatment Fffects of FAFH

Table 6 presents the estimated potential outcome means (POMs) and the average treatment effects of different levels of frequency of having FAFH on energy and macronutrients balance, controlling for covariates. The control level is FAFH consumption at a frequency less than or equal to 3 times per month. The results show that the estimated POMs of the control level are the smallest except for carbohydrate (percentage deviation of carbohydrate). That is, energy, 3 essential nutrients, protein, and fat intakes are the closest to the reference intake levels for individuals who have at-home foods. This means having FAFH generally increases the intake deviation from the reference levels. Interestingly, having FAFH 5–7 times per week results in the second most balanced dietary intakes. For energy intakes, having FAFH 1–4 times per week increases the deviation by 1.62%p on average in comparison to the control level. For 3 essential nutrients (protein, fat, and carbohydrate) intakes, having FAFH 1–4 times per week increases the deviation the most (3.65%p) whereas having FAFH 5–7 times per week increases the least (2.46%p). 

Protein, fat and carbohydrate intakes of at-home eaters deviate from their reference point by on average 33.68%, 39.76%, and 28.85%, respectively. Protein and carbohydrate intake deviations are increased the most by FAFH 1–4 times per week eaters (8.42%p and 2.02%p) whereas fat intake deviation is the highest for individuals consuming FAFH at least 2 times per day (41.22%). The POMs of carbohydrate intakes are lower for treatment levels 2 and 3 than the control level and heavy FAFH consumers are the most balanced in terms of carbohydrate intakes (28.3%).

Table 7 presents the average treatment effects of FAFH on the macrominerals of calcium, sodium, and potassium. For calcium and potassium, intakes of at-home foods eaters deviate the most (43.78% and 35.43%, respectively) from the reference intakes. Having FAFH has positive effects on the balanced intakes of calcium and potassium. Having FAFH 1–4 times per week decreases the deviation by the most (3.14%p for calcium and 2.84%p for potassium). 

The deviations of sodium intakes are relatively larger than that of other nutrients. On average, sodium intakes are 99.9% away from the adequate intake level for those who have foods at home. As the frequency of FAFH increases, the deviation of sodium intake also increases greatly. Having 1–4 times per week increases sodium intakes deviation by 27.97%p and having FAFH 5–7 times per week and at least twice per day increases the deviation by nearly 32%p for both. 

Table 8 presents the estimated average treatment effects of FAFH on body weight outcomes. The outcome in the first column of Table 8 is the deviation of energy intakes from the reference intakes without taking the absolute value. It can be observed that the energy intakes of individuals who have FAFH are higher than that of those who have at-home foods. On average, daily calorie intakes of individuals who have food at home are 9.41% less than their reference point. Having away-from-home foods 1–4 times per week increases the calorie intake by 9.02%p whereas having FAFH 5–7 times per week and at least twice per day increases the intake by 7.68%p and 6.3%p, respectively.

From the multivalued treatment effect analysis, curvilinear patterns are found for BMI and the probability of being obese. Consuming FAFH 5–7 times per week yields the best outcome for BMI (23.50) and the probability of being obese (29.17%). However, individuals who consume FAFH 1–4 times per week have higher BMI (23.74) and the probability of being obese (31.66%) than 5–7 times per week consumers. As in the previous literature, rare FAFH may be perceived as special events and this makes people consume more fatty and sweet food products. Having food at home is associated with 0.0539 higher BMI and 0.62%p higher probability of being obese compared to having FAFH 1–4 times per week. For high-frequency FAFH consumers (consuming at least 2 times per day), BMI and the probability of being obese are the highest. This suggests that the optimal level of having FAFH is found to be 5–7 times per week in terms of BMI and the probability of being obese. 

Additional treatment effect analysis is performed for obesity by age groups (Table 9). The probability of being obese increases as age in the group gets older. The potential outcome means of the control group for young adults (age <40) is 26.99% whereas that for the age group 40 years or more is 34.81% and that for old adults (age ≤50) is 38.43%. Having FAFH at least twice per day significantly increases the probability of being obese by 7.95%p, in comparison to the control level, for the age group 40 years or more. For young adults (age <40), having FAFH seems to decrease the chance of being obese but the average treatment effects are not statistically significant.

## 5. Discussion

The estimation results show that FAFH generally increases the deviation of dietary intakes from each reference point except for carbohydrate, calcium, and potassium and the effects are relatively large for protein and sodium intakes. These results indicate that effects of having FAFH on the deviation of nutrition intake are non-linear, however, its marginal effects do not appear to change from a negative to a positive one. Therefore, no conclusion on optimal frequency of having FAFH in terms of nutrition intake can be drawn. This indicates that food at home are more balanced in terms of energy, three essential nutrients, and sodium intakes. However, this outcome concerns the health policy makers because the dietary intakes deviates substantially for at-home eaters already. Since having a balanced diet is an important key to maintaining good health, it is suggested that all types of restaurants provide nutritional information so consumers can consume food bundles with more balanced nutrition levels. In this sense, there is a need to expand the mandatory nutrition labeling. 

Although FAFH increases caloric intakes, having FAFH 1–4 times per week seems to reduce BMI and the probability of being obese. Additionally, consuming FAFH 5–7 times per week reduces them even more, which implies that the marginal effect of having FAFH is non-linear. The evidence of non-linearity suggests that having FAFH 5–7 times per week is optimum in terms of reducing obesity. However, there are concerns for high-frequency FAFH consumers (individuals who have FAFH at least twice per day) since BMI and the probability of being obese increase greatly. Heavy FAFH consumers who are 40 years old or older are more susceptible to adverse health outcomes with the probability of being obese being 42.76% and 7.95 %p higher in comparison to a hypothetical control group. Respondents who are male, young, unmarried, or live in single households are more likely to consume away-from-home foods at least twice per day. Therefore, policy makers should target these individuals when creating policy with the intention of improving health outcomes for its population. 

## 6. Conclusions

Changes in demographic and socioeconomic characteristics have contributed to an increase in away-from-home foods consumption. This has caused concerns for the consumers’ health as at-home foods are generally believed to be healthier than away-from-home foods. In this paper, inverse probability weighted regression adjustment, a doubly robust method, is applied to the sixth Korean National Health and Nutrition Examination Survey (KNHANES VI) dataset to estimate the effects of different levels of frequency of having FAFH on the balanced dietary intakes and obesity. Unlike previous studies that only consider the effects of not having or having FAFH (binary treatment effect), this paper employs a multivalued treatment effect analysis based on frequency of FAFH consumption, which provides a higher level of detail to policy makers. This study contributes to existing research about FAFH on health outcomes through this multivalued analysis. The empirical model applied in this study allows the non-linear relationship between the frequency of FAFH and health outcomes, and as a result, we are able to identify an optimal level of FAFH consumption frequency for the degree of obesity and deviation of nutrition intake. Since multivalued treatment effects are identified by comparing each treatment with the control group, selection bias is avoided, and the significance of the treatment effects across different levels of treatment is obtained. The latter provides us with evidence that can lead to more insightful and targeted policy design for each hypothetically treated consumer groups.

In summary, the overall results in this study provide evidence that there is no linear relationship between the frequency of having FAFH and the health outcomes of balanced nutrition intake and obesity. This result indicates that policy that intends to enhance consumers’ health should be more elaborate. In other words, the results suggest a potential need to design different policies for different consumer groups. For example, policy efforts should be made to reduce away-from-home consumption of individuals who eat out more often than the optimal level, while policies that reduce away-from-home consumption of individuals who eat out less frequently than the optimum level might not be needed.

This research provides insights into the effects of each different level of having FAFH on the balanced dietary intakes and obesity among adults and thus contributes to existing literature. Based on the research findings, it is expected that health policy makers will be able to develop a healthier diet plan.

## Figures and Tables

**Table 1:** Description of multivalued hypothetical treatment variable.

Variable	Description
FAFH	More than or equal to twice a day = 3, 5–7 times per week = 2, 1–4 times per week = 1, Less than or equal to 3 times per month = 0

Note: Delivery and take-out foods are included, and provisions of meals are excluded in FAFH category.

**Table 2:** Descriptive statistics of outcome variables.

Variable	Description	Mean	Std. Dev.	Min	Max
Energy deviation	(Daily energy intake—EER)/EER	−0.02	0.35	−0.83	2.85
Percentage deviation of energy	Absolute value of ((Daily energy intake—EER)/EER)	0.27	0.22	0.0001	2.85
Percentage deviation of protein	Absolute value of ((Daily protein intake—RI)/RI)	0.41	0.43	2.07 × 10^−5^	6.28
Percentage deviation of fat	Absolute value of ((Daily fat intake-AMDR)/AMDR)	0.41	0.32	3.60 × 10^−6^	5.82
Percengate deviation of carbohydarate	Absolute value of ((Daily carbohydrate intake—AMDR)/AMDR)	0.30	0.26	1.42 × 10^−5^	2.23
Percentage deviation of 3 essential nutrients	Average of percentage deviation of protein, fat and carbohydrate	0.37	0.28	0.02	4.63
Percentage deviation of sodium	Absolute value of ((Daily sodium intake—AI)/AI)	1.26	1.10	0.0001	13.00
Percentage of calcium	Absolute value of ((Daily calcium intake—RI)/RI)	0.42	0.22	4.26 × 10^−5^	2.16
Percentage deviation of Potassium	Absolute value of ((Daily potassium intake—AI)/AI)	0.34	0.23	4.25 × 10^−5^	2.58
BMI	Body mass index	23.68	3.53	14.59	48.96
Obesity	If BMI is greater than or equal to 25 kg/m^2^ = 1, otherwise = 0	0.31	0.46	0	1

Notes: (1) EER: Estimated Energy Requirement; (2) RI: Recommended Intake; (3) AI: Adequate Intake; (4) AMDR: Acceptable Macronutrient Distribution Ranges (55% for carbohydrate and 20% for fat are applied).

**Table 3:** Descriptive statistics of covariates.

Variable	Description	Mean	Std. Dev.	Min	Max
male	Male = 1, Female = 0	0.38	0.49	0	1
age	Age	43.76	12.75	19	64
urban	Urban = 1, Rural = 0	0.47	0.50	0	1
education	At least university = 1, otherwise = 0	0.40	0.49	0	1
cfam	The number of family member(s)	3.24	1.17	1	6
singlehh	If single household = 1, otherwise = 0	0.06	0.24	0	1
ainc	Average monthly household income (unit: 10,000 won) If less than or equal to 17 = 17, 18-1499 = continuous value, If greater than or equal to 1500 = 1500	415.78	284.71	17	1500
married	If married = 1, otherwise = 0	0.80	0.40	0	1
child	If have child under 19 = 1, otherwise = 0	0.42	0.49	0	1
fulltime	If have full-time job = 1, otherwise = 0	0.19	0.39	0	1
temporary	If have temporary job = 1, otherwise = 0	0.24	0.43	0	1
unemployed	If unemployed = 1, otherwise = 0	0.57	0.49	0	1
pedu_uni	Father’s education level If higher than high school graduation = 1, otherwise = 0	0.14	0.35	0	1
medu_uni	Mother’s education level If higher than high school graduation = 1, otherwise = 0	0.06	0.24	0	1
healthy	Subjective health status If more than or equal to “good” = 1, otherwise = 0	0.33	0.47	0	1
activitylim	If have activity limitation = 1, otherwise = 0	0.06	0.23	0	1
body	Subjective body perception If fat = 1, otherwise = 0	0.46	0.50	0	1
drinking	Frequency of drinking If drink twice or more per week = 1, otherwise = 0	0.21	0.41	0	1
stress	Stress awareness If get stress a lot = 1, otherwise = 0	0.26	0.44	0	1
highBP	If high blood pressure = 1, otherwise = 0	0.20	0.40	0	1
hypertension	If hypertension = 1, otherwise = 0	0.24	0.43	0	1
labeluse	If use nutrition label = 1, otherwise = 0	0.31	0.46	0	1
mealdaily	The number of meals per day	2.53	0.56	0	6
strength	The number of days of weight training exercises per week	0.78	1.50	0	5
stretch	The number of days of flexibility exercise per week	1.91	1.96	0	5
dietetictherapy	If practice dietetic therapy =1, otherwise = 0	0.24	0.43	0	1
year13	If year 2013 = 1, otherwise = 0	0.36	0.48	0	1
year14	If year 2014 = 1, otherwise = 0	0.32	0.47	0	1
year15	If year 2015 = 1, otherwise = 0	0.32	0.47	0	1

**Table 4:** Estimation results for the treatment model by Multinomial logit.

Variables	1–4 Times per Week	5–7 Times per Week	At Least Twice per Day
male	0.3577 ***	1.4795 ***	1.8656 ***
	(0.0831)	(0.0898)	(0.1287)
age	−0.0369 ***	−0.0475 ***	−0.0502 ***
	(0.0045)	(0.0052)	(0.0074)
urban	0.0858	0.1755 **	0.1985 *
	(0.0648)	(0.0749)	(0.1085)
education	0.4987 ***	0.4076 ***	0.3938 ***
	(0.0793)	(0.0883)	(0.1223)
cfam	−0.1409 ***	−0.1302 ***	−0.1680 **
	(0.0390)	(0.0447)	(0.0664)
singlehh	0.0343	0.2573	0.6158 **
	(0.1580)	(0.1820)	(0.2430)
ainc	0.0011 ***	0.0015 ***	0.0015 ***
	(0.0001)	(0.0002)	(0.0002)
married	0.0975	−0.2368	−0.8527 ***
	(0.1480)	(0.1614)	(0.2122)
child	0.1284	0.2015 *	0.1964
	(0.0928)	(0.1058)	(0.1541)
fulltime	0.1713	2.0289 ***	1.9568 ***
	(0.1294)	(0.1263)	(0.1584)
temporary	0.1316	1.2096 ***	0.6755 ***
	(0.0801)	(0.0886)	(0.1383)
pedu_uni	0.1975	0.1861	0.3476 **
	(0.1253)	(0.1376)	(0.1760)
medu_uni	0.4360 *	0.4863 **	0.1983
	(0.2264)	(0.2381)	(0.2831)
healthy	0.2863 ***	0.3059 ***	0.1307
	(0.0738)	(0.0836)	(0.1198)
activitylim	−0.3813 ***	−0.580 ***	−0.3086
	(0.1228)	(0.1650)	(0.2439)
body	0.1159 *	0.1314 *	0.2704 **
	(0.0660)	(0.0767)	(0.1119)
drinking	0.3389 ***	0.4428 ***	0.8488 ***
	(0.0929)	(0.1004)	(0.1307)
stress	−0.0360	0.1710 *	0.2911 **
	(0.0777)	(0.0878)	(0.1227)
highBP	−0.1816 **	−0.0864	−0.5131 ***
	(0.0904)	(0.1068)	(0.1696)
prehyper	−0.1251	−0.1689 *	−0.0857
	(0.0827)	(0.0959)	(0.1334)
labeluse	−0.0816	−0.1669 *	−0.1383
	(0.0735)	(0.0858)	(0.1253)
mealdaily	−0.1478 **	0.0008	0.1130
	(0.0615)	(0.0711)	(0.1026)
year13	−0.0476	−0.0862	−0.2004
	(0.0796)	(0.0919)	(0.1333)
year14	−0.1517 *	−0.1019	−0.1093
	(0.0805)	(0.0928)	(0.1328)
Constant	2.2025 ***	0.7760 **	−0.4771
	(0.2666)	(0.3013)	(0.4259)
Observations	7456		

Note: (1) Standard errors are shown in parentheses. ***, **, and * indicate coefficients are significant at the 0.01, 0.05 and 0.1 levels, respectively. (2) The base outcome is having Food Away From Home less than or equal to three times per month.

**Table 5:** The baseline means for control and hypothetically treated groups.

Covariates	Control (Having FAFH Less Than or Equal to 3 Times per Month)	Treated 1 (Having FAFH 1–4 Times per Week)	Treated 2 (Having FAFH 5–7 Times per Week)	Treated 3 (Having FAFH at Least Twice per Day)
male	0.22	0.27	0.58	0.68
age	50.14	43.37	40.74	38.30
urban	0.45	0.47	0.49	0.50
education	0.21	0.42	0.49	0.49
cfam	3.09	3.28	3.33	3.18
singlehh	0.06	0.05	0.06	0.11
ainc	331.49	422.15	463.09	451.03
married	0.92	0.82	0.73	0.59
child	0.34	0.44	0.45	0.38
fulltime	0.06	0.10	0.36	0.38
temporary	0.20	0.21	0.30	0.22
pedu_uni	0.07	0.15	0.17	0.21
medu_uni	0.02	0.06	0.08	0.09
healthy	0.24	0.35	0.38	0.35
activitylim	0.11	0.05	0.03	0.05
body	0.47	0.47	0.45	0.47
drinking	0.14	0.18	0.26	0.34
stress	0.24	0.24	0.28	0.32
highBP	0.27	0.17	0.19	0.14
prehyper	0.24	0.22	0.24	0.30
labeluse	0.29	0.35	0.28	0.27
mealdaily	2.60	2.50	2.52	2.50
year13	0.36	0.36	0.36	0.33
year14	0.34	0.31	0.32	0.33

**Table 6:** The average treatment effects on energy and macronutrients balance.

POMs/ATEs	Precentage Deviation of Energy	Percentage Deviation of Three Essential Nutrients	Percentage Deviation of Protein	Percentage Deviation of Fat	Percentae Deviation of Carbohydarate
POMs					
0	0.2515 ***	0.3409 ***	0.3368 ***	0.3976 ***	0.2885 ***
	(0.0070)	(0.0068)	(0.0100)	(0.0085)	(0.0078)
1	0.2677 ***	0.3774 ***	0.4210 ***	0.4026 ***	0.3087 ***
	(0.0045)	(0.0056)	(0.0087)	(0.0061)	(0.0052)
2	0.2632 ***	0.3656 ***	0.4100 ***	0.3990 ***	0.2877 ***
	(0.0059)	(0.0073)	(0.0108)	(0.0083)	(0.0069)
3	0.2737 ***	0.3695 ***	0.4133 ***	0.4122 ***	0.2830 ***
	(0.0137)	(0.0145)	(0.0210)	(0.0174)	(0.0160)
ATEs					
1 vs. 0	0.0162 *	0.0365 ***	0.0842 ***	0.0051	0.0202 **
	(0.0083)	(0.0088)	(0.0132)	(0.0104)	(0.0094)
2 vs. 0	0.0117	0.0246 **	0.0732 ***	0.0014	−0.0008
	(0.0091)	(0.0099)	(0.0147)	(0.0119)	(0.0104)
3 vs. 0	0.0222	0.0286 *	0.0765 ***	0.0146	−0.0054
	(0.0154)	(0.0160)	(0.0232)	(0.0193)	(0.0178)

Notes: Robust standard errors in parentheses. ***, **, and * indicate coefficients are significant at the 0.01, 0.05 and 0.1 levels, respectively. POM and ATE denote potential-outcome mean and average treatment effect, respectively.

**Table 7:** The average treatment effects on macrominerals balance.

POMs/ATEs	Percentage Deviation of Calcium	Percentage Deviation of Sodium	Percentage Deviation of Potassium
POMs			
0	0.4378 ***	0.9990 ***	0.3543 ***
	(0.0086)	(0.0314)	(0.0076)
1	0.4064 ***	1.2787 ***	0.3259 ***
	(0.0043)	(0.0214)	(0.0046)
2	0.4155 ***	1.3166 ***	0.3327 ***
	(0.0058)	(0.0256)	(0.0061)
3	0.4101 ***	1.3161 ***	0.3278 ***
	(0.0142)	(0.0616)	(0.0129)
ATEs			
1 vs. 0	−0.0314 ***	0.2797 ***	−0.0284 ***
	(0.0096)	(0.0378)	(0.0089)
2 vs. 0	−0.0223 **	0.3176 ***	−0.0216 **
	(0.0103)	(0.0403)	(0.0098)
3 vs. 0	−0.0277 *	0.3172 ***	−0.0264 *
	(0.0165)	(0.0690)	(0.0150)

Notes: Robust standard errors in parentheses. ***, **, and * indicate coefficients are significant at the 0.01, 0.05 and 0.1 levels, respectively. POM and ATE denote potential-outcome mean and average treatment effect, respectively.

**Table 8:** The average treatment effects on calorie intake, BMI and obesity.

POMs/ATEs	Energy Deviation	BMI	Obesity
POMs			
0	−0.0941 ***	23.7889 ***	0.3228 ***
	(0.0102)	(0.0863)	(0.0109)
1	−0.0039	23.7350 ***	0.3166 ***
	(0.0069)	(0.0575)	(0.0080)
2	−0.0173 **	23.4991 ***	0.2917 ***
	(0.0088)	(0.0736)	(0.0107)
3	−0.0311	24.0698 ***	0.3622 ***
	(0.0206)	(0.1731)	(0.0243)
**ATEs**			
1 vs. 0	0.0902 ***	−0.0539	−0.0062
	(0.0122)	(0.0943)	(0.0125)
2 vs. 0	0.0768 ***	−0.2898 ***	−0.0311 **
	(0.0133)	(0.1052)	(0.0144)
3 vs. 0	0.0630 ***	0.2809	0.0394
	(0.0230)	(0.1887)	(0.0261)

Note: Robust standard errors in parentheses. *** and ** indicate coefficients are significant at the 0.01 and 0.05 levels, respectively. POM and ATE denote potential-outcome mean and average treatment effect, respectively.

**Table 9:** The average treatment effects on obesity for different age groups.

POMs/ATEs	Obesity Age < 40	Obesity Age ≥ 40	Obesity Age ≥ 50
POMs			
0	0.2699 ***	0.3481 ***	0.3843 ***
	(0.0213)	(0.0132)	(0.0146)
1	0.2631 ***	0.3457 ***	0.3723 ***
	(0.0116)	(0.0108)	(0.0134)
2	0.2615 ***	0.3193 ***	0.3512 ***
	(0.0140)	(0.0154)	(0.0213)
3	0.2621 ***	0.4276 ***	0.4362 ***
	(0.0345)	(0.0315)	(0.0383)
ATEs			
1 vs. 0	−0.00676	−0.0024	−0.0120
	(0.0229)	(0.0159)	(0.0181)
2 vs. 0	−0.00842	−0.0288	−0.0331
	(0.0241)	(0.0192)	(0.0245)
3 vs. 0	−0.00776	0.0795 **	0.0519
	(0.0397)	(0.0336)	(0.0403)

Notes: Robust standard errors in parentheses. *** and ** indicate coefficients are significant at the 0.01 and 0.05 levels, respectively. POM and ATE denote potential-outcome mean and average treatment effect, respectively.

## Supplementary Materials

The following are available online at https://www.mdpi.com/1660-4601/17/2/586/s1, Table S1: Korean Dietary Reference Intakes, Table S2: Covariate balance summary for treated group 1. Table S3: Covariate balance summary for treated group 2. Table S4: Covariate balance summary for treated group 3. Table S5: Mean estimation of outcome variable over FAFH.
