# Supplementary material for: Eating Out and Consumers’ Health: Evidence on Obesity and Balanced Nutrition Intakes
Source: Int J Environ Res Public Health. 2020 Jan 16;17(2):586. doi: 10.3390/ijerph17020586 (PMC7014261; doi:10.3390/ijerph17020586)
Supplement: Supplementary file 1 [file ijerph-17-00586-s001.pdf]

International Journal of
Environmental Research and Public Health

MDPI

Supplementary Material

Table S1. Korean dietary reference intakes.

Sex	Age	Energy (EER) kcal/day	Protein (RI) g/day	Fat (AMDR) %	Carbohydrate (AMDR) %	Calcium (RI) mg/day	Sodium (AI) mg/day	Potassium (AI) mg/day
Male	19-29	2,600	65	15-30	55-65	800	1500	3500
	30-49	2,400	60	15-30	55-65	800	1500	3500
	50-64	2,200	60	15-30	55-65	750	1500	3500
Female	19-29	2,100	55	15-30	55-65	700	1500	3500
	30-49	1,900	50	15-30	55-65	700	1500	3500
	50-64	1,800	50	15-30	55-65	800	1500	3500

Source: 2015 Korean Dietary Reference Intakes, Ministry of Health and Welfare

Note: (1) EER: Estimated Energy Requirement
(2) RI: Recommended Intake
(3) AI: Adequate Intake
(4) AMDR: Acceptable Macronutrient Distribution Ranges

Table S2. Covariate balance summary for treated group 1.

Covariates	Standardized Differences		Variance Ratio	
	Raw	Weighted	Raw	Weighted
male	0.1289	-0.0059	1.1678	0.9976
age	-0.5657	0.0106	1.2477	0.9300
urban	0.0515	0.0768	1.0077	1.0137
education	0.4580	0.0486	1.4475	1.0239
cfam	0.1581	-0.0852	0.8961	0.9997
singlehh	-0.0530	0.0739	0.8134	1.3343
ainc	0.3330	-0.1252	1.1525	0.6253
married	-0.2907	-0.0086	1.9666	1.0112
child	0.2157	0.0061	1.1009	1.0025
fulltime	0.1547	0.0712	1.6616	1.1248
temporary	0.0349	-0.0103	1.0517	0.9879
pedu_uni	0.2542	-0.0459	1.9322	0.9178
medu_uni	0.2353	-0.0553	3.4635	0.8339
healthy	0.2575	-0.0807	1.2673	0.9531
activitylim	-0.2184	-0.0120	0.4926	0.9563
body	-0.0082	-0.0139	0.9988	0.9975
drinking	0.1315	0.0487	1.2783	1.0752
stress	0.0061	0.0652	1.0072	1.0757

highBP	-0.2434	0.0009	0.7174	1.0015
prehyper	-0.0634	-0.0275	0.9220	0.9680
labeluse	0.1242	-0.0171	1.1032	0.9857
mealdaily	-0.1927	-0.0116	1.1116	1.0227
year13	0.0111	-0.0067	1.0061	0.9959
year14	-0.0567	0.0469	0.9590	1.0377

Table S3. Covariate balance summary for treated group 2.

Covariates	Standardized Differences		Variance Ratio	
	Raw	Weighted	Raw	Weighted
male	0.7921	0.0038	1.4467	1.0015
age	-0.7996	-0.0034	1.1681	0.8644
urban	0.0805	0.1099	1.0099	1.0159
education	0.5972	0.0592	1.4823	1.0285
cfam	0.1979	-0.0835	0.9438	1.0136
singlehh	-0.0077	0.0683	0.9721	1.3075
ainc	0.4739	-0.1140	1.2401	0.6035
married	-0.5169	0.0071	2.6696	0.9908
child	0.2206	-0.0207	1.1022	0.9911
fulltime	0.8136	0.0789	4.4112	1.1381
temporary	0.2404	0.0150	1.3226	1.0173
pedu_uni	0.3179	-0.0409	2.1762	0.9268
medu_uni	0.3035	-0.0389	4.4315	0.8822
healthy	0.3178	-0.0663	1.3102	0.9621
activitylim	-0.3106	-0.0057	0.3087	0.9796
body	-0.0510	-0.0080	0.9916	0.9988
drinking	0.3181	0.0510	1.6435	1.0780
stress	0.1005	0.0391	1.1160	1.0462
highBP	-0.1956	0.0096	0.7766	1.0151
prehyper	0.0000	-0.0344	0.9999	0.9598
labeluse	-0.0168	-0.0449	0.9838	0.9622
mealdaily	-0.1485	-0.0416	1.0207	0.9083
year13	-0.0023	0.0053	0.9985	1.0033
year14	-0.0528	0.0046	0.9621	1.0038

Table S4. balance summary for treated group 3.

Covariates	Standardized Differences		Variance Ratio	
	Raw	Weighted	Raw	Weighted
male	1.0653	-0.0283	1.2836	0.9883
age	-0.9981	0.1869	1.2097	0.9766
urban	0.0931	0.0246	1.0117	1.0056
education	0.5978	-0.0266	1.4844	0.9849
cfam	0.0753	-0.0224	1.0756	1.1644
singlehh	0.1703	0.0013	1.6808	1.0055
ainc	0.4409	-0.1958	1.1357	0.5049
married	-0.8365	0.0279	3.2817	0.9640
child	0.0786	-0.0479	1.0490	0.9775
fulltime	0.8606	0.0380	4.5196	1.0670
temporary	0.0603	0.0173	1.0903	1.0199
pedu_uni	0.3973	-0.0515	2.4769	0.9078
medu_uni	0.3345	-0.0939	4.9091	0.7237
healthy	0.2503	-0.0475	1.2636	0.9736
activitylim	-0.2412	-0.0834	0.4459	0.7152
body	-0.0084	0.0580	1.0002	1.0054
drinking	0.4988	0.0265	1.9204	1.0408
stress	0.1875	0.1337	1.2044	1.1479
highBP	-0.3240	0.0960	0.6167	1.1473
prehyper	0.1185	0.0734	1.1321	1.0801
labeluse	-0.0317	-0.0840	0.9707	0.9266
mealdaily	-0.1771	0.0040	1.1033	0.9546
year13	-0.0582	0.0768	0.9640	1.0422
year14	-0.0280	0.0533	0.9817	1.0413

International Journal of
Environmental Research and Public Health

MDPI

Table S5. Mean estimation of outcome variables over FAFH.

FAFH	Percentage deviation of energy	Percentage deviation of protein	Percentage deviation of fat	Percentage deviation of carbohydarate	Percentage deviation of calcium	Percentage deviation of sodium	Percentage deviation of potassium	Energy deviation	BMI	obesity
0	0.257***	0.326***	0.437***	0.294***	0.471***	0.899***	0.365***	-0.117***	23.93***	0.332***
	(0.006)	(0.007)	(0.007)	(0.006)	(0.006)	(0.021)	(0.006)	(0.007)	(0.086)	(0.011)
1	0.269***	0.426***	0.401***	0.314***	0.403***	1.252***	0.327***	-0.009	23.49***	0.289***
	(0.004)	(0.008)	(0.006)	(0.005)	(0.004)	(0.019)	(0.004)	(0.006)	(0.066)	(0.008)
2	0.264***	0.438***	0.398***	0.287***	0.400***	1.459***	0.329***	0.010	23.62***	0.311***
	(0.005)	(0.010)	(0.007)	(0.006)	(0.005)	(0.025)	(0.005)	(0.007)	(0.0724)	(0.010)
3	0.279***	0.485***	0.411***	0.277***	0.384***	1.653***	0.323***	0.046***	24.14***	0.364***
	(0.011)	(0.021)	(0.015)	(0.011)	(0.009)	(0.055)	(0.010)	(0.016)	(0.155)	(0.021)

Note: Standard errors in parentheses. ***, **, and * indicate coefficients are significant at the 0.01, 0.05 and 0.1 levels, respectively.

International Journal of
Environmental Research and Public Health
